# Digital health literacy and well-being of health sciences students from MU-Sofia during the pandemic

**DOI:** 10.1093/eurpub/ckac129.713

**Published:** 2022-10-25

**Authors:** N Leventi, A Vodenicharova, K Popova

**Affiliations:** 1 Department of Health Technologies Assessment, Medical University – Sofia, Sofia, Bulgaria; 2 Department of Bioethics, Medical University – Sofia, Sofia, Bulgaria; 3 Department of Social Medicine, Medical University – Sofia, Sofia, Bulgaria

## Abstract

**Background:**

The aim of the study is to demonstrate the Digital health literacy and the well-being of the students from different faculties in Medical University - Sofia during the pandemic.

**Methods:**

To achieve the purpose of the study a web-based questionnaire was distributed among health sciences students from the Faculty of Public Health and the Medical College -Sofia, as well as medical students from the Faculty of Medicine all from Medical University -Sofia in Bulgaria. Data was collected between February and April 2022, and all respondents participated anonymously and voluntarily. Established statistical methods were used in data analysis.

**Results:**

Completed questionnaires were received from 239 students. Data collected show that among participants the majority (81,4%) were females, and 73,3% were studying in a Bachelor's programme. Among the respondents 87.7% found it easy, or very easy to use the proper words or search query to find the information they were looking for about coronavirus or related topics. Finally 29,3% of health sciences students expressed low to very low well-being during the last two weeks and the rest 70,7% expressed high well-being.

**Conclusions:**

The presented results draw attention to the fact that during the pandemic health sciences students demonstrate the appropriate skills in searching and acquiring the information about coronavirus or related topics. In addition, translating and applying the information could contribute to benefit the psychological well-being of the students. In a digitally transformed health sector it is significant for future health professionals to obtain competencies including digital health literacy to promote health and well-being of the patients and provide better outcomes for them. The necessity of digital competences was underlined and thus we need further more, and in depth education in ICT, and digital technologies to all students, starting from the beginning of their studies.

